# Supplementary material for: Sirtuin5 contributes to colorectal carcinogenesis by enhancing glutaminolysis in a deglutarylation-dependent manner
Source: Nat Commun. 2018 Feb 7;9:545. doi: 10.1038/s41467-018-02951-4 (PMC5803207; doi:10.1038/s41467-018-02951-4)
Supplement: Supplementary file 1 — Supplementary Information [file 41467_2018_2951_MOESM1_ESM.pdf]

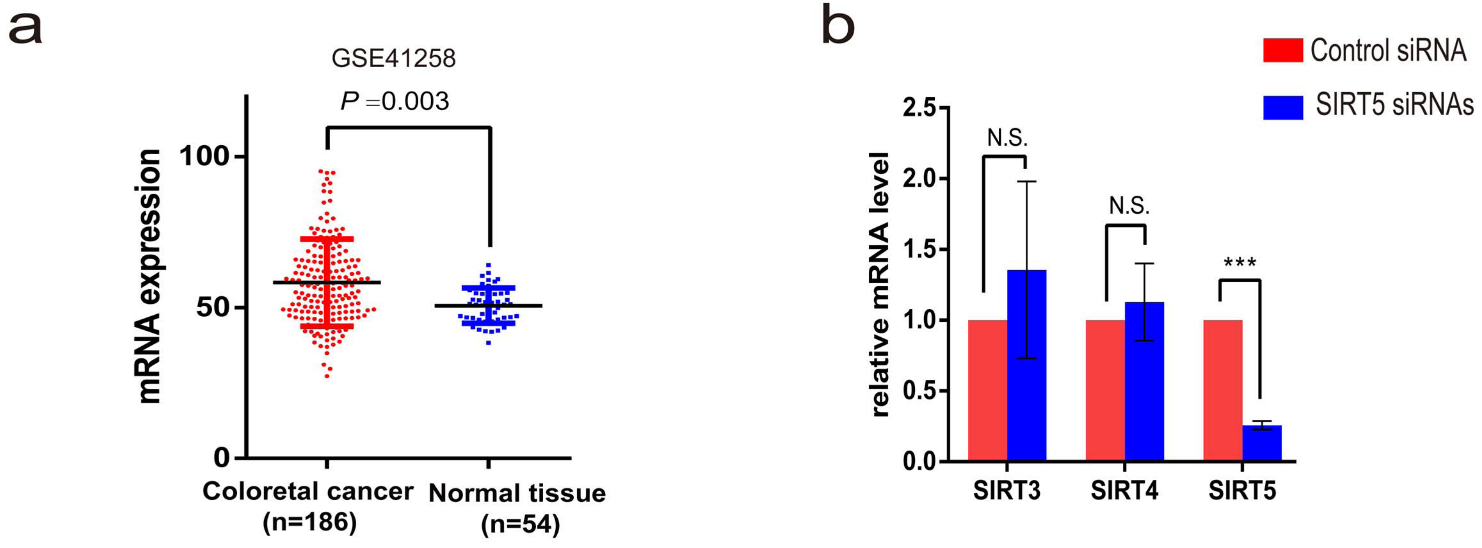

Supplementary Figure 1.(a)The mRNA expression of *SIRT5* was extracted from GEO data set GSE41258. The comparison between human CRC (n=186) and normal tissues (n=54) was performed using Student's t-test. Data are presented as the mean ± SD.The *P*-values are indicated. (b)Quantitative real-time reverse transcription PCR (qRT-PCR) assay to measure the levels of three mitochondrial sirtuins (*SIRT3*, *SIRT4*, and *SIRT5*) in the absence or presence of *SIRT5* siRNAs. Data are presented as the mean ± SD of three independent samples.Student's t-test.****P*<0.001, N.S. = not significant for the indicated comparison.

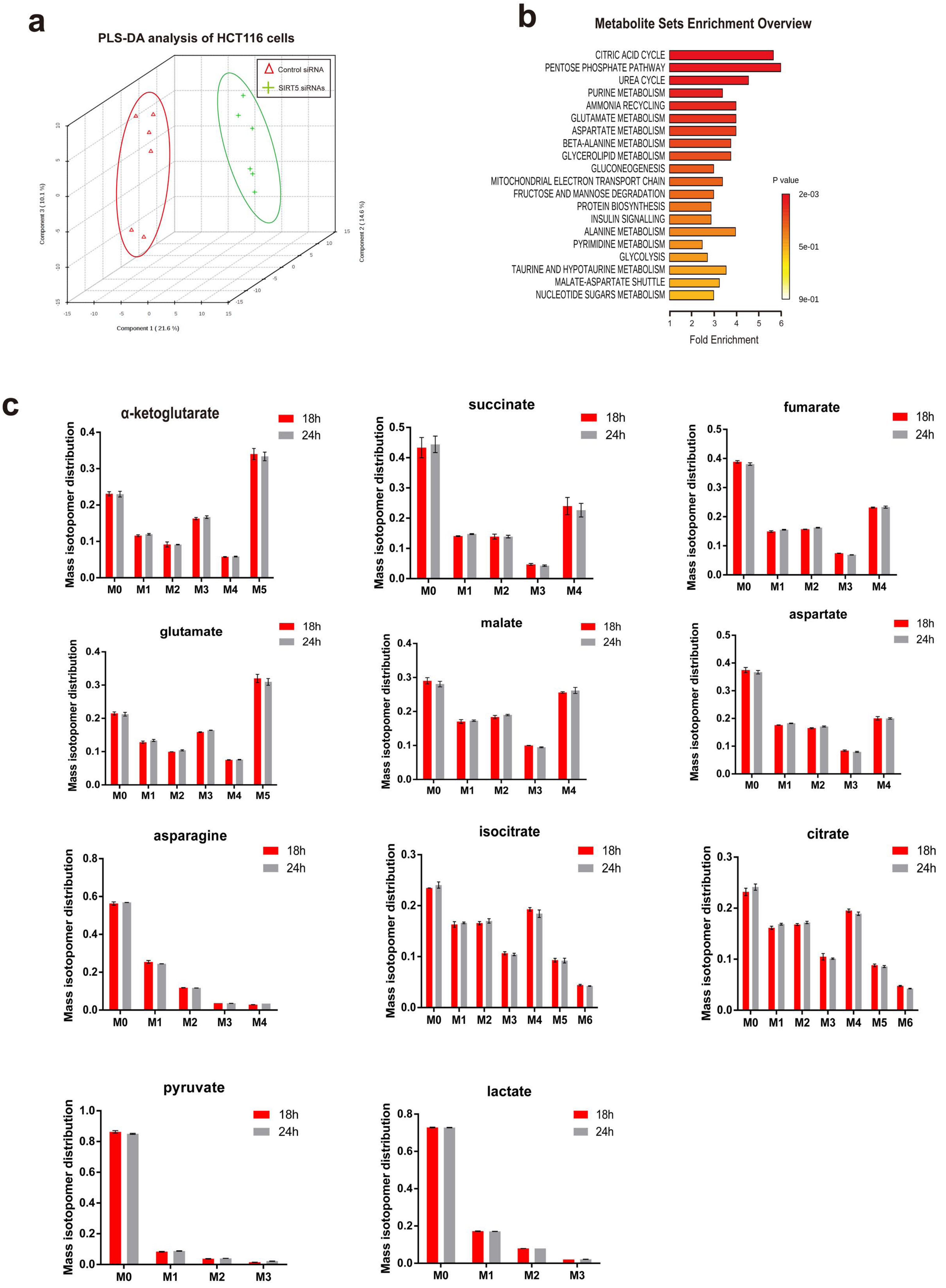

Supplementary Figure 2.(a) 3-D score plot of the differentially abundant metabolites classified by performing partial least squares discriminant analysis (PLS-DA) in HCT116 cells transfected with *SIRT5* siRNAs or control siRNA. n= 6 samples for each condition. The PLS-DA model enables the correct classification of the samples based on the different abundances of metabolites, which allows identification of the most significant metabolites that explain the different effects of the treatments. (b) Metabolite set enrichment of the differentially abundant metabolites determined by gas chromatography-mass spectrometry (GC-MS) from HCT116 cells transfected with *SIRT5* siRNAs or control siRNA.(c) Mass isotopomer distributions (MIDs) of metabolites extracted from LoVo cells at 18 and 24h after [U-$^{13}C_5$] glutamine incubation, metabolites were detected by GC-MS and corrected for natural abundance. Metabolite labeling did not significantly change over the course of 18 to 24 h, indicating that the cells have achieved metabolic and isotopic steady state at the indicated time-point. Data are the mean ± SD of three independent samples.

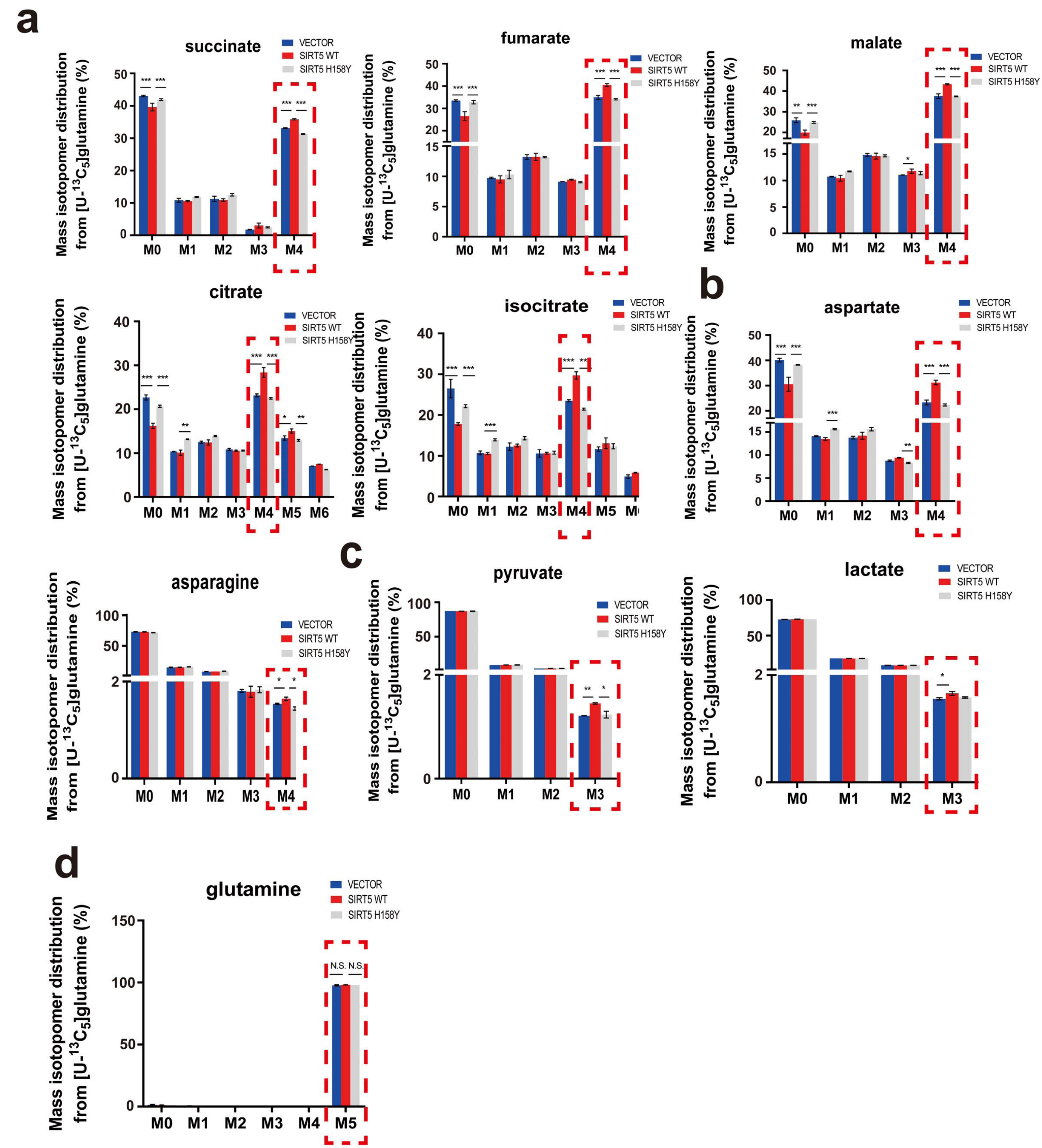

Supplementary Figure 3.(a)LoVo cells stably expressing the control vector, SIRT5 WT, and SIRT5 H158Y were cultured with [U-$^{13}C_5$] glutamine for 24h. MIDs of TCA cycle metabolites extracted from cells were quantified by GC-MS.(b,c) MIDs of aspartate, asparagine (b) and pyruvate, lactate (c) derived from glutamine were quantified by GC-MS.(d) Measured MIDs of glutamine in LoVo cells. The fraction of m+5 glutamine indicates cellular uptake of glutamine. MIDs were corrected for natural abundance. Data in a-d are the mean ± SD of three independent samples. ANOVA with Tukey's test.**P*< 0.05, ***P*< 0.01, ****P*< 0.001. N.S. = not significant for the indicated comparison.

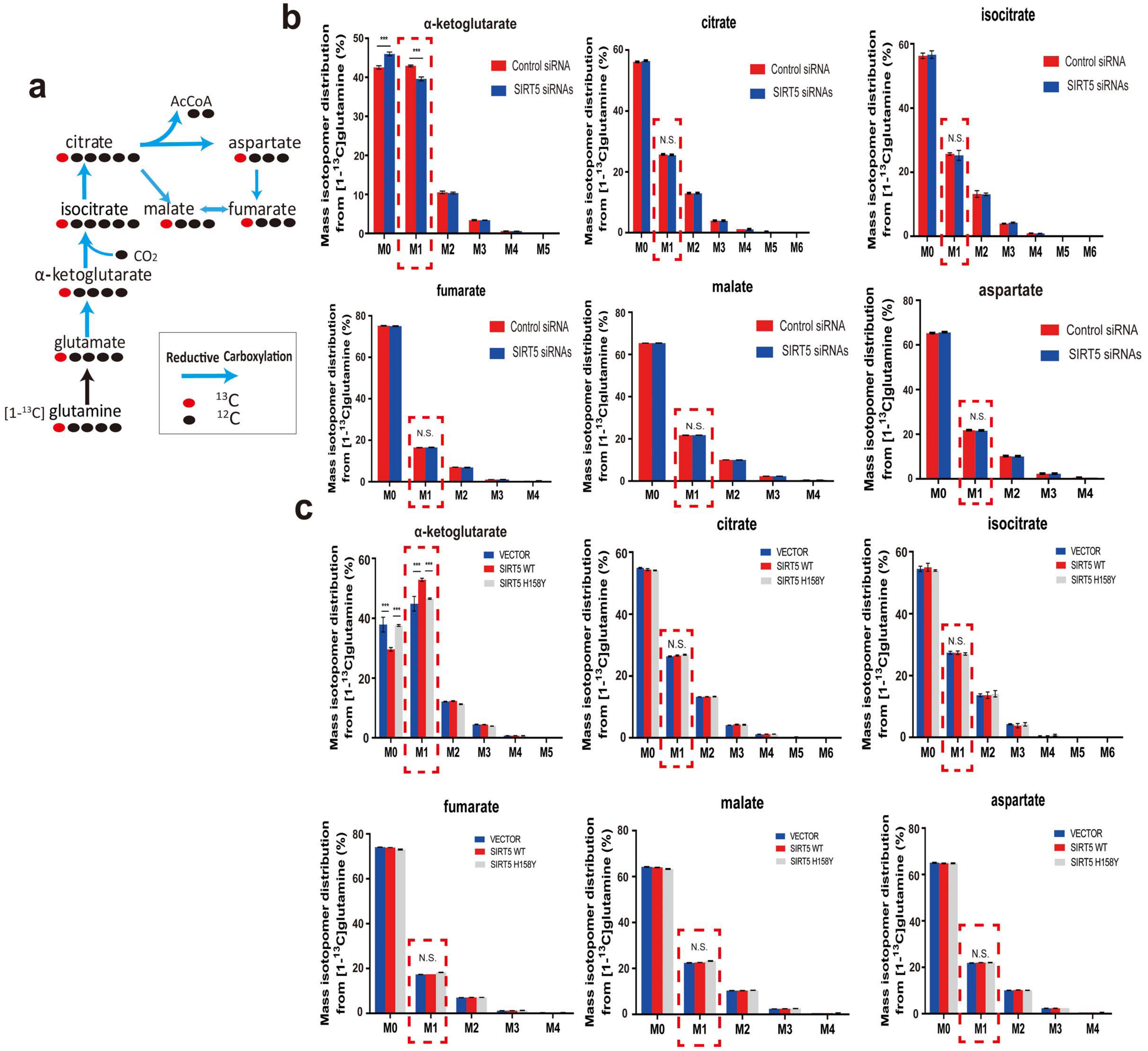

Supplementary Figure 4. (a) Carbon atom transition map depicting the labeling patterns of metabolites derived from [1-^{13}C] glutamine through the reductive metabolism pathway. Red circles represent carbons derived from [1-^{13}C] glutamine, and black circles are unlabeled. The blue arrows indicate reductive carboxylation flux from glutamine.(b)Mass isotopologues of α-KG, fumarate, malate, citrate, isocitrate, and aspartate in SIRT5-silenced LoVo cells after culture with [1-^{13}C] glutamine for 24 h. Data are shown as the mean ± SD. n=4. Student's t-test. ****P*< 0.001. N.S. = not significant for the indicated comparison.(c) Mass isotopologues of α-KG, fumarate, malate, citrate, isocitrate, and aspartate in LoVo cells stably expressing control vector, SIRT5 WT, and SIRT5 H158Y after culture with [1-^{13}C] glutamine for 24 h. Data are the mean ± SD of three independent samples. *P* values were calculated by ANOVA with Tukey's test. ****P*< 0.001. N.S. = not significant for the indicated comparison.

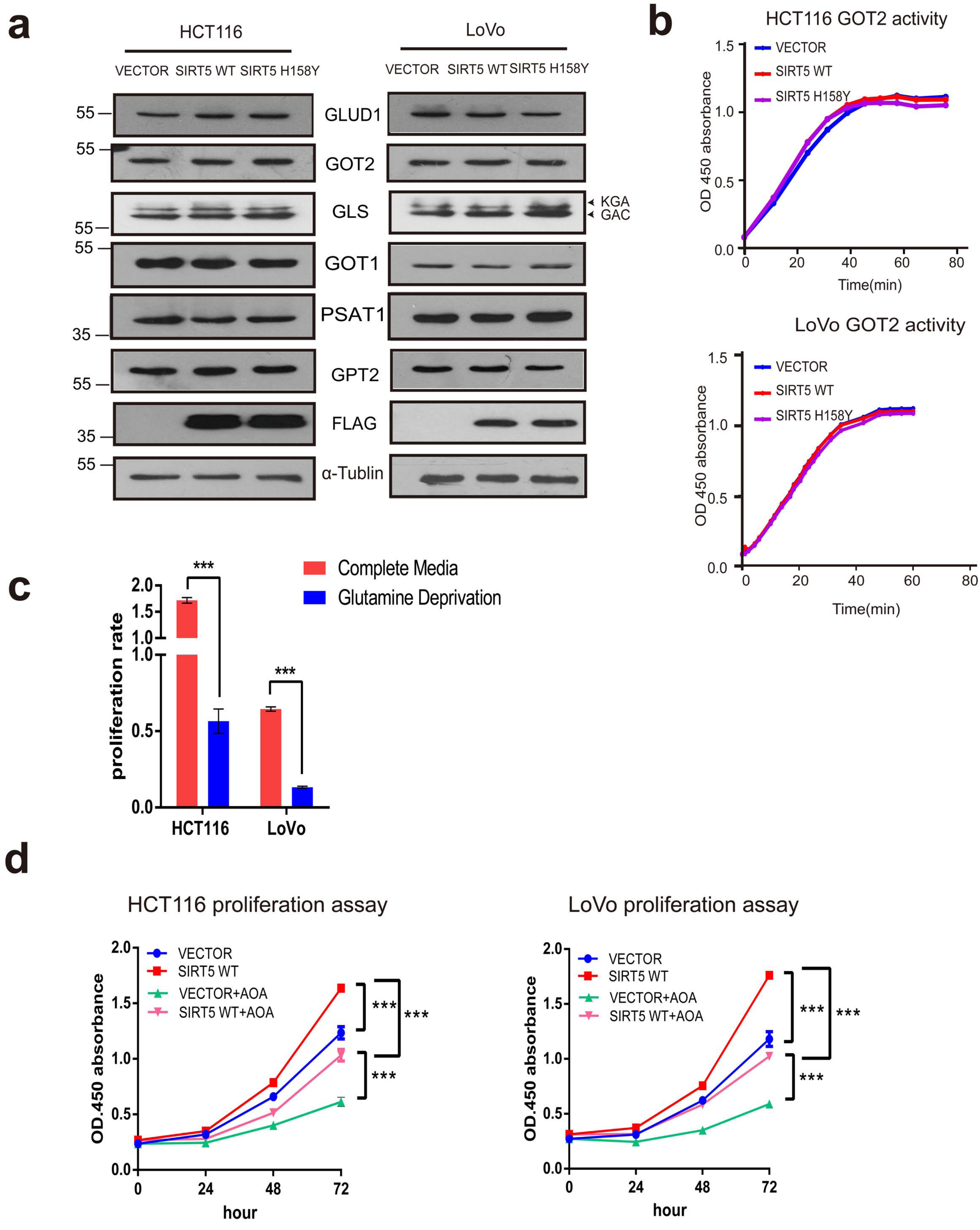

Supplementary Figure 5.(a)Western blot showing that SIRT5 WT/H158Y mutation did not change the GLUD1, GOT1/2, GLS (including kidney-type glutaminase (KGA isoform), and glutaminase C(GAC isoform)), PSAT1, and GPT2 protein levels in CRC cells. α-Tublin served as the loading control.(b) Representative tracings (n = 3) of GOT2 enzyme activity in HCT116 and LoVo cells stably expressing the control vector, SIRT5 WT, or SIRT5 H158Y. The experiments were repeated three times. (c) Proliferation rate of HCT116 and LoVo cells stably expressing the control vector or SIRT5 WT plasmid in complete media or glutamine deprived media. Data are the mean ± SD of four independent samples from a representative experiment. Student's t-test. ***P<0.001.(d) CCK-8 assays of HCT116 and LoVo cells stably expressing the control vector or SIRT5 WT treated with or without the pan-transaminases inhibitor AOA (0.5 mM). ANOVA with Tukey's test. Data are the mean ± SD of five independent samples. ***P<0.001.

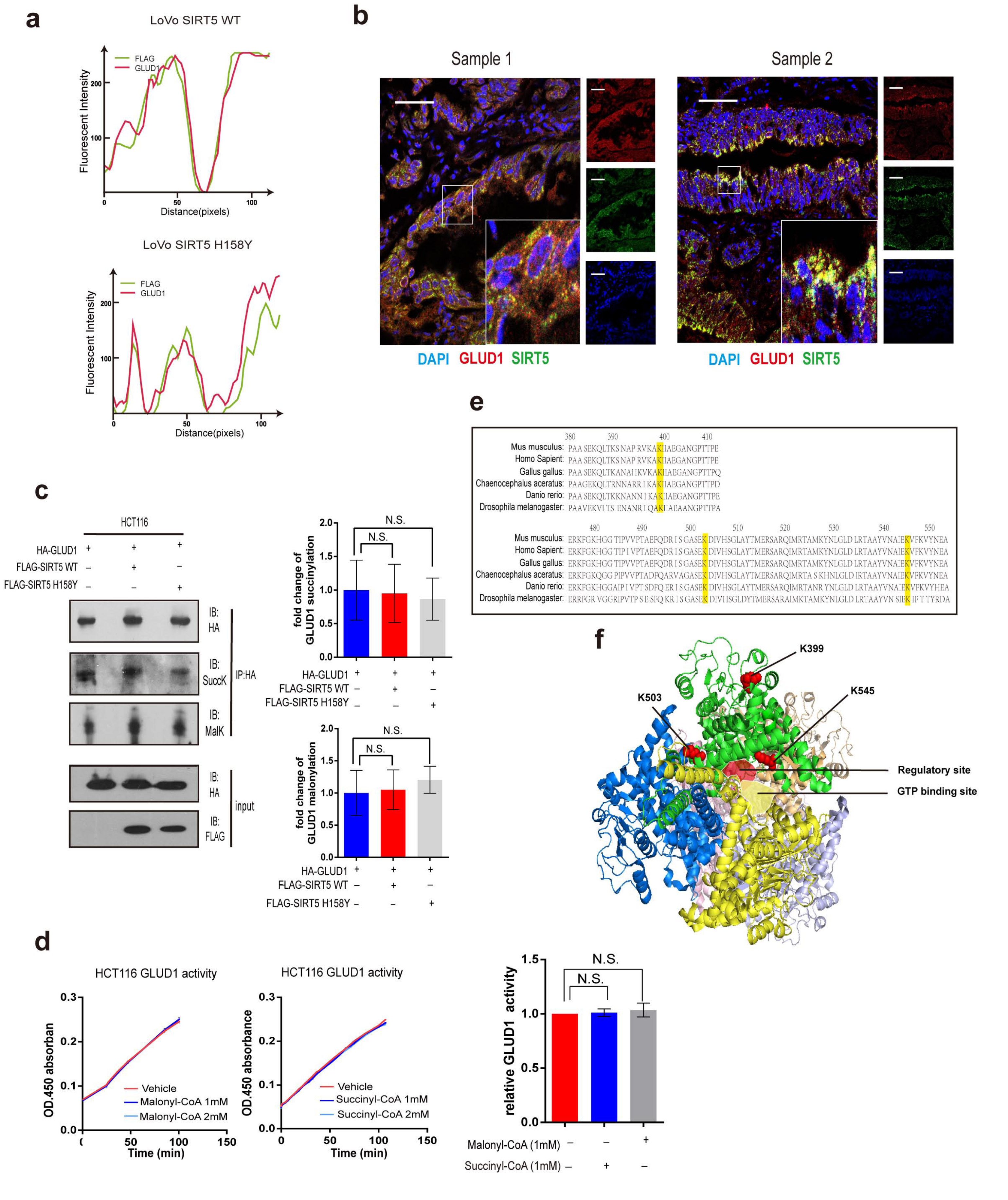

Supplementary Figure 6. (a) Fluorescence intensity of FLAG-SIRT5 (green line) and GLUD1 (red line) traced along the white line in LoVo cells using the line profiling function of the Image J software.(b)Immunofluorescence analysis of SIRT5 (green) and GLUD1 (red) expression in CRC tissues; yellow in the merged magnified images (left) indicates SIRT5 and GLUD1 co-localization. Scale bars indicate 50 μm.(c) Exogenous GLUD1 proteins were purified by immunoprecipitation in HCT116 cells expressing the control vector, SIRT5 WT, and SIRT5 H158Y. Succinylation (SuccK) and malonylation levels (MalK) of GLUD1 were determined by western blotting. Integrated density values were calculated using Image J. The results are the mean ± SD of two independent experiments, and are shown relative to the vector control. ANOVA with Tukey's test. N.S. = not significant for the indicated comparison.(d)HA-Tagged GLUD1 proteins were purified and incubated with different concentrations of succinyl- or malonyl-CoA (0, 1, and 2 mM) at 37°C for 60min. The GLUD1 enzyme activity was then determined. Left, representative images (n=3). Right, quantification of GLUD1 activity. The results are shown as the mean ± SD. ANOVA with Tukey's test. N.S. = not significant for the indicated comparison.(e) Sequence alignment of GLUD1 revealed that three lysine residues(K399, K503, and K545) that could be glutarylated are conserved across different species, including human (*Homo sapiens*, NCBI reference number: NP_005262.1), mouse (*Mus musculus*, NP_032159.1), chicken(*Gallus gallus*, XP_015143519.1), blackfinicefish(*Chaenocephalus aceratus*,UniProtKB_P82264), zebrafish (*Danio rerio*, NP_955839.2), and fruitfly(*Drosophila melanogaster*, NP_996274.1).(f)Structure and quaternary conformation of human GLUD1 (PDB ID: 1L1F) simulated from the protein data bank. Each subunit is represented by a different color. Lysine residues (K399, K503, and K545) are indicated with red marks. The GTP binding (yellow) and regulatory (red) sites in one protomer are shown.

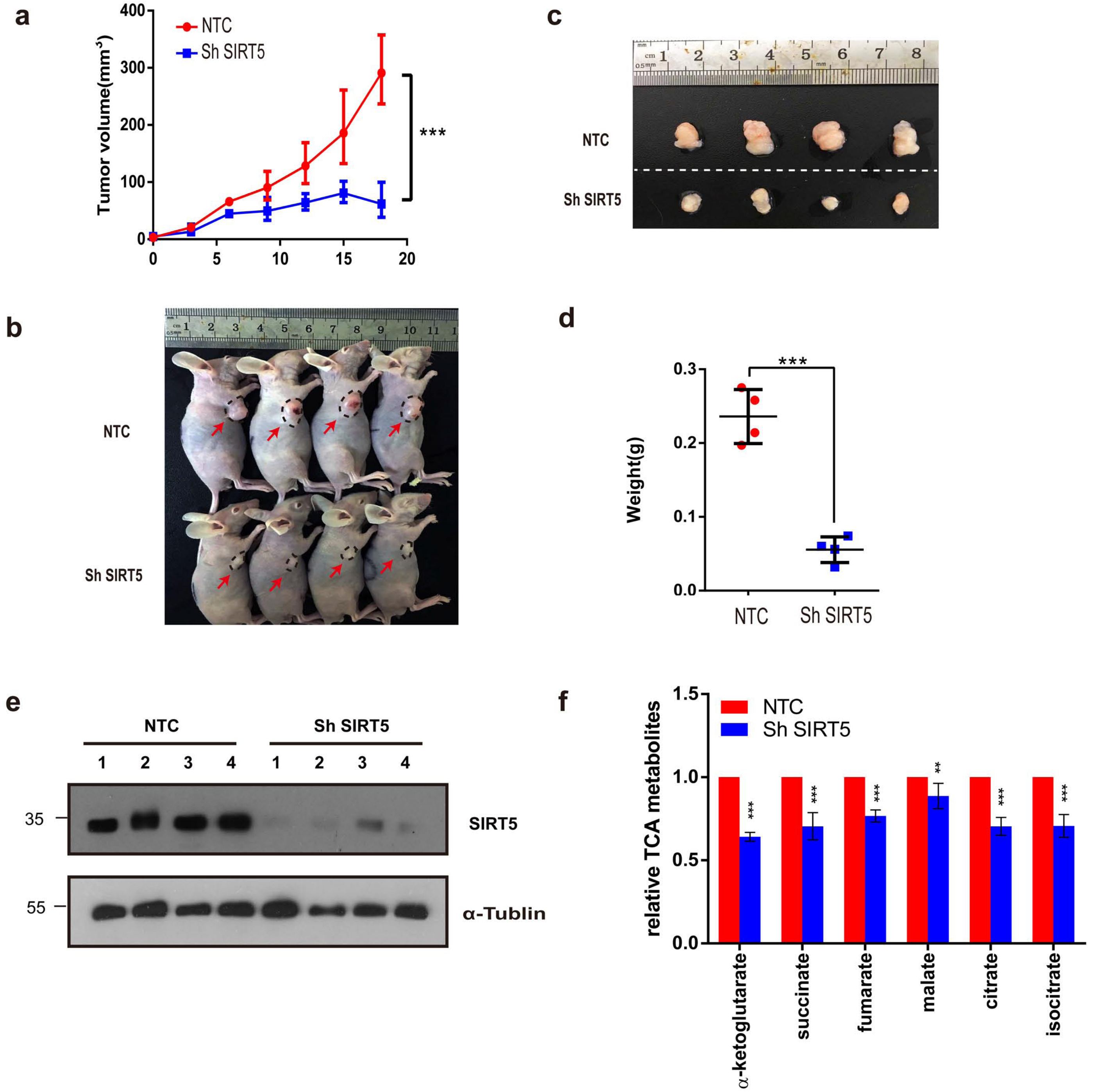

Supplementary Figure 7. (a) HCT116 cells stably expressing the non-target control (NTC) shRNA or *SIRT5* shRNA were injected subcutaneously into nude mice (n=4 for each group). Tumor volumes were measured at the indicated time points and the mean tumor volumes were calculated. Student's t-test. ***$P< 0.001$.(b-d) At the end of experiment, tumors from the two groups were dissected, photographed (b,c), and weighted. Each dot represents the tumor mass from one mouse (d); n=4 for each group. Student's t-test. ***$P < 0.001$.(e)SIRT5 protein levels and the knockdown efficiency in xenografts were confirmed by immunoblotting.(f)The abundance of α-KG, succinate, fumarate, malate, citrate and isocitrate in tumor lysates were quantified by GC-MS analysis. Student's t-test. ***$P< 0.001$. The results in a, d, f are shown as the mean ± SD.

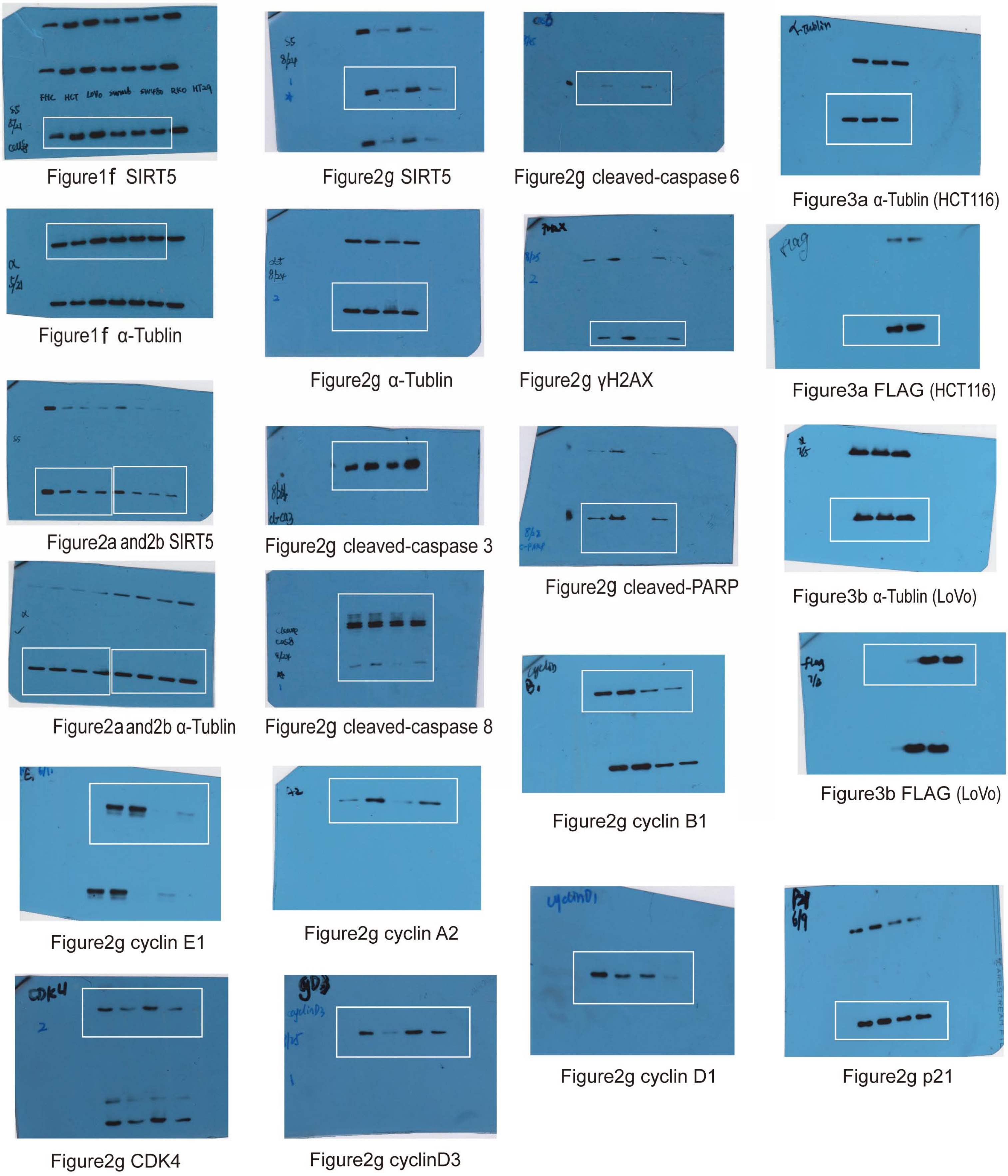

Supplementary Figure 8.Uncropped scans of western blots presented in Figure 1,2 and 3.

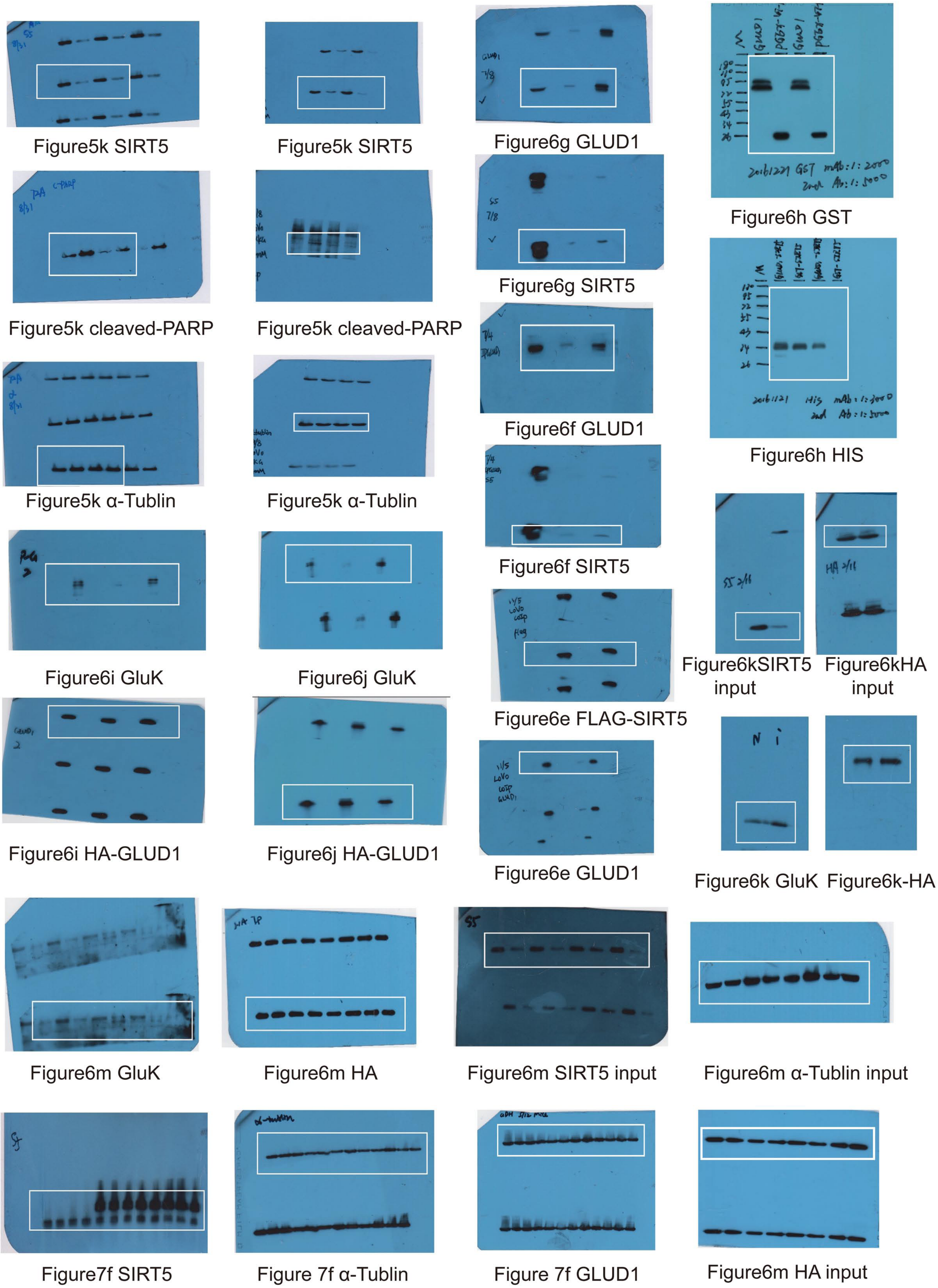

Supplementary Figure 9. Uncropped scans of western blots presented in Figure 5, 6 and 7.

SUPPLEMENTARY TABLES

Supplementary Table 1. Clinicopathological features of SIRT5 expression in patients with CRC

Clinicopathological features	Total	SIRT5 expression				
		low		high		*P*-value
		No. of patients (%)		No. of patients (%)		
**Age (years)**						
≤ 65	40	20	50	20	50	**0.846**
> 65	47	25	52.1	23	47.9	
**Gender**						
Male	46	24	52.2	22	47.8	**0.839**
Female	42	21	50	21	50	
**Histological grade**						
I, I–II, II	75	40	53.3	35	46.7	**0.322**
II–III, III, III–IV	13	5	38.5	8	61.5	
**Tumor size (cm^3)**						
< 20	30	20	66.7	10	33.3	**0.036***
≥ 20	57	25	43.9	32	56.1	
**T stage**						
T1+T2+T3	62	36	58.1	26	41.9	**0.038***
T4	26	9	34.6	17	65.4	
**Lymph node metastasis**						
Absent	58	35	60.3	23	39.7	**0.016***
Present	30	10	33.3	20	66.6	
**Distant metastasis**						
Absent	85	44	51.8	41	48.2	**0.53**
Present	3	1	33.3	2	66.7	
**AJCC stage**						
1-2	56	35	62.5	21	37.5	**0.005***
3-4	32	10	31.3	22	68.8	

* Chi-square test was used for all comparisons and *P* values less than 0.05 were accepted as statistically significant.

Supplementary Table 2. List of metabolites found to be differentially regulated upon *SIRT5* knockdown

Compounds-M.T. (min)	VIP	*P*-value	Fold Change
cholesterol-35.2847	1.234862	0.043841	0.10494
xanthosine-26.2209	1.718701	0.00166	0.000421
uracil-9.95633	1.907835	0.000158	2.3822
trehalose-6-phosphate-26.7863	1.297544	0.03231	0.56504
threonic acid-13.9835	2.211334	2.60E-09	1.5803
taurine-24.9349	1.25529	0.039796	2.491
succinic acid-9.52320	2.088121	2.82E-06	0.73654
stearic acid-27.2551	1.404573	0.018031	0.60599
scopoletin-8.12380	1.338511	0.026103	5.5117
ribulose-5-phosphate-21.6267	1.358399	0.023434	0.25338
prostaglandin A2-35.2049	1.519094	0.008682	0.60455
phosphate-8.99122	1.452343	0.013495	2.746
phloroglucinol-25.1636	1.246637	0.041474	20.189
palmitoleic acid-23.9170	1.935538	0.0001	4.75E-05
palmitic acid-24.3803	1.363547	0.022778	0.65635
O-phosphonothreonine-10.777	1.580557	0.005539	1.7632
N-ethylglycine-7.94414	1.661498	0.002839	0.000145
myristic Acid-20.4625	1.687964	0.002231	0.5213
L-malic acid-12.8651	1.824888	0.000507	0.795919
L-4-hydroxyphenylglycine-31.0229	1.99215	3.43E-05	12.211
inosine 5'-monophosphate-24.5585	1.37623	0.021221	0.19532
heptadecanoic acid-25.956	1.61386	0.004256	0.48048
guanosine-31.8341	1.80781	0.000626	1.5799
glycocyamine-14.8205	1.754645	0.001146	6.8403
glycerol-29.7549	1.850201	0.000365	0.033515
glucose-6-phosphate-28.0456	1.325286	0.028	0.6125
galactonic acid-22.6369	2.049631	8.80E-06	1.7758
fumaric acid-10.1864	1.609727	0.004402	0.846491
Fructose 2,6-biphosphate degr -17.3424	1.439389	0.014627	0.36851
ethyl cinnamate-14.3353	1.459894	0.012866	2.7611
erythrose 4-phosphate-23.2727	1.696271	0.002063	9.67E-05
D-talose-21.6717	1.820421	0.000536	0.37393
dithioerythritol-9.69934	1.402539	0.018247	0.62219
diglycerol-29.4922	2.257292	3.55E-14	5.20E-05
D-fructose 1,6-bisphosphate-29.0498	1.853348	0.00035	0.59965
citric acid-19.7808	2.114867	1.08E-06	0.671272
beta-mannosylglycerate-22.9889	1.589579	0.005165	0.45066
beta-glutamic acid-13.3293	1.235691	0.043671	1.6805
asparagine-7.60792	1.733432	0.001431	0.49548
alpha-ketoglutaric acid-14.1984	1.300221	0.031874	0.33185

allo-inositol-23.5860	2.042224	1.07E-05	1.8825
adenosine 5-monophosphate-34.9312	1.365912	0.022481	0.48234
adenine-20.7409	1.885077	0.000223	1.5238
aconitic Acid-18.3703	2.072042	4.66E-06	0.727932
6-phosphogluconic acid-28.6589	1.682535	0.002347	0.48437
5-aminoimidazole-4-carboxamide-7.45383	1.475213	0.01166	0.61763
5,6-dihydrouracil-12.7036	1.700998	0.001972	1.5439
4-nitrocatechol-21.4643	1.770195	0.000967	0.11501
3-phosphoglycerate-17.9796	1.959888	6.48E-05	2.4178
3-hydroxybutyric acid-13.1618	1.673736	0.002544	0.1971
1-hexadecanol-22.6925	1.939937	9.28E-05	0.000124

Significantly different metabolites were identified by a combination of two methods: $P < 0.05$ by t-tests, and Variable Importance in Projection (VIP) >1 by partial least squares discriminant analysis (PLS-DA).

Supplementary Table 3. Summary of previously identified lysine acylation of enzymes involved in glutamine metabolism

			lysine modifications (sites)		
Reference	sample source	protein	Ksucc	Kmal	Kglu
Park J, 2014[23]	mouse MEF	GLUD1	147, 183, 211, 390, 503, 527, 545		
		GLS	135, 126		
Du J, 2011[21]	bovine liver	GLUD1	84, 109, 162,363, 414, 457, 503, 527	503, 457, 527	
		GOT2	309		
Rardin MJ, 2013[22]	mouse liver	GLUD1	480, 90, 457, 211, 365, 545, 363, 346, 527, 162, 110, 548, 503, 84, 187, 415, 68, 200, 191		
		GOT2	185, 73, 309, 296, 227, 396, 159, 345, 234, 313, 302, 333, 90, 404, 122, 387		
Tan M, 2014[25]	mouse liver	GLUD1			503, 527, 84, 90, 390, 363, 399, 545, 480
		GOT2			90, 59, 302, 296, 234, 227, 82, 404, 73, 363, 122, 309, 338, 396
Nishida Y, 2015[24]	mouse liver	GLUD1		90,84, 346, 503	
		GOT2		296, 338, 404	
Colak G, 2015[34]	Human fibroblasts	GLS		245	
Weinert BT,2013[33]	E. coli	GLUD	16, 184, 276		
		GOT	116, 37, 42, 51, 276, 236, 134, 50		
	human Hela cells	GLUD1	191, 503, 211,183, 346, 527, 457, 545, 415		
		GLS	164, 311		
	Mouse liver	GLUD1	187, 181, 211, 84, 200, 503, 457, 545, 548, 527, 162, 352, 415, 390, 90		
		GOT2	90, 122, 302, 309, 73, 82, 159, 404, 234, 338, 345, 396, 227, 363, 185		
		GLS	244, 329, 515, 172		

Abbreviation: Ksucc: Lysine succinylation, Kglu: Lysine glutarylation, Kmal: Lysine malonylation
